# Propagation of Adult SSCs: From Mouse to Human

**DOI:** 10.1155/2013/384734

**Published:** 2013-01-01

**Authors:** Laura A. Martin, Marco Seandel

**Affiliations:** Department of Surgery, Weill Cornell Medical College, 1300 York Avenue, LC-701, P.O. Box 282, New York, NY 10065, USA

## Abstract

Adult spermatogonial stem cells (SSCs) represent a distinctive source of stem cells in mammals for several reasons. First, by giving rise to spermatogenesis, SSCs are responsible for the propagation of a father's genetic material. As such, autologous SSCs have been considered for treatment of infertility and other purposes, including correction of inherited disorders. Second, adult spermatogonia can spontaneously produce embryonic-like stem cells *in vitro*, which could be used as an alternative for therapeutic, diagnostic, or drug discovery strategies for humans. Therefore, an increasing urgency is driving efforts to understand the biology of SSCs and improve techniques to manipulate them *in vitro* as a prerequisite to achieve the aforementioned goals. The characterization of adult SSCs also requires reproducible methods to isolate and maintain them in long-term culture. Herein, we describe recent major advances and challenges in propagation of adult SSCs from mice and humans during the past few years, including the use of unique cell surface markers and defined cultured conditions.

## 1. Introduction

The spermatogonial stem cells (SSCs) of the adult testis in mammals possess both the extraordinary ability to self-renew, in order to maintain a near life-long pool of stem cells and the means to differentiate into lineage-committed germ cells. Adult SSCs, however, represent a unique model for a number of reasons. First, male fertility and genetic diversity of species both depend on continuous, normal spermatogenesis during reproductive life [1, 2]. Second, spermatogonia *in vitro *can spontaneously produce embryonic-like stem cells [3] and therefore can be used as a model to study mechanisms of reprogramming and maintenance of pluripotency or to develop strategies for regenerative therapy for humans similar to embryonic stem cells (ES) or induced-pluripotent stem cells (iPS). The use of spermatogonial-derived pluripotent stem cells could avoid ethical concerns over the use of ES cells and also obviate the need for exogenous pluripotent factors, such as those necessary to generate iPS from adult somatic cells [4]. Furthermore, the likelihood of immunological rejection by the host would be greatly reduced compared to ES-derived cells, since autologous pluripotent cells can potentially be isolated from the human testis [5, 6]. In addition, SSCs represent one of the few adult stem cell populations that can be maintained in a long-term *in vitro *culture system [7, 8] and for which a functional *in vivo *transplantation assay has been established [9, 10]. However, the identification and further characterization of the true stem cells among the spermatogonia remain challenging because of the absence of specific markers that would allow the isolation and further analysis of this population. This is particularly true in humans, because human germ cells tend to be technically difficult to study. Additionally, the unique features of adult SSCs as opposed to neonatal SSCs are important to consider, since spermatogonia undergo substantial changes in gene expression during the post-natal period (e.g., reduction of OCT4 expression) and also in function [11–13]. This review will focus on the theoretical and practical basis for long-term culture of adult mouse SSCs and recent efforts toward the development of human SSC cultures.

## 2. SSC Identity and the Spermatogonial Stem Cell Niche

Spermatogenesis consists of the differentiation of male germ cells into spermatozoa, the male gametes that carry genetic information to subsequent generations and occur within the seminiferous tubules [14, 15]. The seminiferous tubules are highly structured convoluted tubules, consisting of a lumen, into which the spermatozoa are released, and the peripheral basement membrane. Two types of somatic cells are located on the periphery of the seminiferous tubules: (1) peritubular myoid cells covering the external side of the basement membrane and (2) Sertoli cells that form the epithelium on the inner surface of the basement membrane to nourish the male germ cells in various stages of maturity. Spermatogenesis in the adult testis relies on SSCs that are derived from prospermatogonia that themselves mature from gonocytes in the fetus. Gonocytes, in turn, are derived from primordial germ cells that migrate into the gonad during embryonic development.

While most recent studies have relied on molecular markers of spermatogonia, it is important to understand the morphological basis for subclassifying this group of undifferentiated germ cells [16]. The spermatogonial population is located along the basement membrane of the seminiferous tubules and has been grouped, based on morphological criteria, into A, Intermediate, and B subtypes. The A type spermatogonia is then divided into undifferentiated (A_undiff_) and differentiated [17]. A_ undiff_ represents the most primitive spermatogonia, and it is characterized by minimal heterochromatin condensation. In the case of rodents, *A*_undiff_ can be further classified into *A*_s_ (single), *A*_pair_ (cohorts of two cells), and *A*_aligned_ (cohorts of 4, 8, and 16 cells) [18–20]. The *A*_s  _ spermatogonia are thought to include the stem cell population, while *A*_pr_ and *A*_aligned_ represent their progeny. *A*_aligned_ continue to mature into differentiated spermatogonia to ultimately produce diploid spermatocytes. In primates, however, *A*_undiff_ spermatogonia are separated uniquely into two subtypes of *A*_s_, *A*_d_ (dark) or *A*_p_ (pale), based on distinct levels of chromatin condensation [21–26]. Type *A*_d_ are considered the reserve stem cells, while *A*_p_ divide symmetrically to produce either new *A*_p_ or type B spermatogonia that will further differentiate to form spermatocytes and spermatids. 

The stem cells represent a minor fraction of the undifferentiated spermatogonial pool. In rodents, it remains controversial whether stem cell capacity resides exclusively in the *A*_s_ pool, or a fraction thereof, or whether *A*_pair_ cells also retain stem cell activity. While we and others have previously referred to undifferentiated spermatogonia as “spermatogonial progenitor cells,” this term is somewhat confusing due to the unintended implication that such cells may be even more primitive than SSCs. The identity of human SSCs is unknown, in part due to the challenge of maintaining them in long term in culture (to be discussed below) [27]. However, it is generally accepted that the human SSCs correspond to a minor fraction of the *A*_d_ or *A*_p_ spermatogonia. Recent studies suggest that there are two functional populations of SSCs in the mouse testis [28, 29]: self-renewing SSCs (referred to as actual stem cells) and another population that maintains the ability to self-renew but only under stressful conditions (referred to as potential stem cells) [30, 31]. These studies seem to support the idea of plasticity within the hierarchy of spermatogonial differentiation in the sense that SSCs comprise a heterogeneous population including cells with different degrees of stem cell potential, whereas certain cells that are committed to differentiation may switch back and self-renew in response to physiological or pathophysiological perturbation. If a similar paradigm applies to humans, then this may conflict directly with one of the central assumptions underlying classical models for the kinetics of maturation of early human spermatogonia; namely, differentiation is linear, unidirectional, and irreversible [24].

One of the most critical elements for stem cell maintenance and function is the associated microenvironment, or niche, that provides physical support and regulates fate decisions of stem cells [31–33]. The niche concept, first proposed by Schofield in 1978 [34], refers to distinct microanatomical locations where tissue-specific stem cells reside. The stem cell niche comprises several components, including resident cells that create essential structural features, provide the proper growth factor milieu to promote self-renewal and/or differentiation of the stem cells, and maintain the stem cell population without excessive proliferation [35]. Spermatogonia are in close contact with Sertoli cells, which are considered one of the most critical constituents of the SSC niche. Sertoli cells exhibit polarity and are connected through tight junctions that create a blood-testis barrier, dividing the epithelium into basal and adluminal compartments. While spermatogonia reside in the basal compartment, germ cells entering meiosis cross the tight junctions and occupy the adluminal zone where subsequent steps of spermatogenesis take place, until the spermatozoa are finally released to the lumen [1, 36–39]. Such subcompartmentalization enables differential exposure of the spermatogonia to signals either secreted by interstitial cells or elaborated by the vascular network, while differentiated germ cells, adluminal to the Sertoli cell tight junctions, are less exposed to such factors. Some data suggest that the vascular network and interstitial tissues also directly contribute to the stem cell niche in the testis [40, 41]. In particular, Leydig cells, best known for producing testosterone, and a subpopulation of peritubular myoid cells may contribute to the function of the SSC niche by secreting specific factors, such as cytokine colony-stimulating factor (CSF1) which potentiates self-renewal in mice cultured spermatogonia.

Some of the more tantalizing questions that arise are whether or not stem cells are immortal or long lived, and whether aging is due, in part, to the progressive cell-autonomous loss of stem cell self-renewal capability, the progressive deterioration of the supporting niche, or perhaps both. SSCs, like hematopoietic and hair follicle stem cells, can be used to address these questions, as such studies require functional assays that are available for only a handful of organ systems [31, 42, 43]. The work by Ryu et al. (2006) using SSC transplantation into a heterologous recipient environment (young or aged, busulfan-treated mouse testis) suggests that SSCs are potentially immortal, since the self-renewal capability of SSCs from an older donor was maintained in a young environment, while aged testes failed to support normal colony formation [44]. Whether extremely long replicative potential is exclusive to SSCs (perhaps because the germline is essential for survival of a species) or whether such longevity is a characteristic shared with other adult stem cells remains unclear. Recent work by Chakkalakal et al. (2012) on the muscle stem cell niche shows that quiescence is essential to maintain stem cell function and that the aged niche disrupts quiescence state of the stem cells, promoting differentiation through an increase in FGF signaling [45]. This effect ultimately leads to depletion of the self-renewing population, supporting the idea that aging of the niche is the root cause of the loss of stem cell capacity in adult tissues. 

## 3. Long-Term Culture of Adult SSCs

The studies discussed previously suggest that microenviromental changes (i.e., niche deterioration) are critical for loss of stem cell maintenance or to drive differentiation over self-renewal and vice versa. The identification of such signals emanating from the SSC niche is therefore critical to establish long-term culture conditions of SSCs. An essential component of the spermatogonial niche is glial cell line-derived neurotrophic factor (GDNF), which is secreted by Sertoli cells [46]. Mutant mice deficient in GDNF exhibit disrupted spermatogenesis and loss of germ cells. In contrast, transgenic mice that overexpress GNDF accumulate undifferentiated spermatogonia, which ultimately lead to tumor-like structures composed of germ cells. Based on these data, preliminary studies defined specific factors and cell culture conditions that increased survival of male mouse germ cells *in vitro* [7, 47–49]. Such signals include GDNF and FGF2 (formerly bFGF); these were combined with SIM mouse embryo-derived thioguanine and ouabain-resistant (STO) feeders that were previously shown to support different stem cell survival [47, 50–52]. Using these tools, Kubota et al. (2004) developed a defined culture system that promoted long-term *in vitro *expansion on SSCs from mouse pup testis [53]. Additionally, two other factors facilitated the successful establishment of long-term cultures. First, donor testis cells were enriched for SSCs by means of surface markers. Second, a serum-free medium was developed, since previous studies had suggested that serum could induce apoptosis or differentiation of SSCs. In fact, this culture system was able to support the expansion of neonatal, pup, and even adult SSCs from several mouse strains, which was the main limitation in previous reports. To date, however, much of the data on SSC culture has been obtained using neonatal testis as the donor tissue as opposed to that of adults, likely because adult SSC lines have been challenging to derive.

Further studies demonstrated that the dependence of SSCs on GDNF signaling is conserved across species, like rat and rabbit, supporting the critical role of GDNF in mammalian SSCs [54–56]. However, GDNF alone is not sufficient to enable long-term culture of SSCs. Moreover, RET and GFR*α*1, the GDNF receptor complex, are expressed in spermatogonia but are not entirely restricted to the stem cells [57–59]. Several studies have shown that in adult mice the fraction of testicular cells expressing GFR*α*1 is not enriched for SSCs, opposite to what is seen in early stages of postnatal development [57]. Additionally, the RET-positive fraction of cells in postnatal mice is not enriched in SSCs. It has been suggested that GDNF is the main survival factor in spermatogonia, while other factors could be responsible for the fate decision during SSC division. Similarly, while FGF2 alone does not support maintenance of SSCs in culture, it does increase the proliferation rate in conjunction with GDNF. The role of FGF2 in the stem cell niche *in vivo* is still unclear, but it is known that various cell types in the testis produce FGF2, including Sertoli, Leydig and differentiating germ cells [60, 61]. Moreover, a recent study shows that FGF2 improves self-renewal of SSCs *in vitro,* activating the MAP2K1 signaling pathway that upregulates ETV and BCL6B, two critical transcription factors for SSCs survival [62, 63]. 

Other extrinsic factors have been shown to enhance the survival of SSCs [46–48, 53]. EGF and IGF-1, for example, seem to have similar effects to FGF2 [49]. On the other hand, CSF1 is expressed in Leydig, cells and a subset of leukocytes and, as mentioned previously, promotes self-renewal of mouse SSC in culture. Furthermore, the CSF1 receptor (CSF1R) is also highly expressed in undifferentiated spermatogonia in mouse testis [41]. However, CSF1 does not increase proliferation in cultures maintained in the presence of GDNF and FGF2, suggesting that CSF1 alters cell fate decisions in SSCs in culture [40]. Finally, although leukemia inhibitory factor (LIF) has a major role in maintaining pluripotency of ES cells and facilitates the establishment of germ cell colonies from the newborn testis, increased proliferation of mouse and rat SSCs was not seen with addition of LIF either to serum-containing media or to GDNF-dependent serum-free cultures [49, 54, 64, 65]. 

The development of systems that facilitate the expansion *in vitro* of SSCs rapidly spawned attempts to specifically culture adult SSCs. The justification for developing adult SSC cultures is several fold. First, the transmission of genetic information to offspring requires faithful spermatogenesis during adulthood and maintenance of a pristine stem cell pool. Also, the adult testis will ultimately be the primary source of human SSCs for *in vitro* genetic manipulation and potentially reparative therapies (i.e., germ line modification). While pluripotent stem cells had been successfully generated previously from neonatal mouse testis, it remained unclear until recently whether the same could be achieved for wild-type adult SSCs in long-term culture [66]. Some systems for derivation and long-term *in vitro* expansion of adult SSCs were inefficient. Moreover, previous methods required the initial enrichment of SSCs using immunoselection (with the caveat that specific markers for SSCs remain still unknown), similar to other adult stem cells, or required cryptorchid mice that contain a higher ratio of stem cells over other types. 

In 2007, we developed a highly proliferative long-term culture system to expand adult SSCs, free of nongermline contaminants [3, 67]. The method used mitotically inactivated testicular stromal cells as feeders, based on the hypothesis that removal of somatic cells from the initial culture disrupts the stem cell niche; this approach allows the *in vitro* propagation of functional SSCs for over a year. SSCs cultured in such conditions self-renew and can reconstitute spermatogenesis after transplantation into busulfan-treated recipients. Furthermore, the *in vitro* milieu preserves the ability of adult SSCs, even after long term in culture, to generate pluripotent adult stem cells that can differentiate into derivatives of the three germ layers and contribute to chimeric embryos. We also identified a novel putative surface marker, an orphan G-protein-coupled receptor (GPR125), which is expressed in the testis exclusively in undifferentiated spermatogonia and can be utilized to track spermatogonia within the mixture of testicular cells. 

Recently, similar approaches utilizing other forms of testicular feeder cell cultures have been utilized to model niche-stem cell interactions [68, 69]. The Shinohara group provided evidence that CXCL12 and GDNF are chemotactic factors that promote homing of SSCs into their niche; this system reproduces the *in vitro *formation of cobblestone colonies growing underneath the stroma similar to previously described colonies in hematopoietic stem cell (HSC) cultures [70, 71]. The cobblestone formation assay has been utilized as an *in vitro* alternative to evaluate HSC potential, particularly useful in certain circumstances when direct transplantation is not possible. It is possible that a similar approach could be utilized with cobblestone colonies derived from germ cells. Nevertheless, the method allows the identification of molecules involved in proliferation and homing of SSCs that can possibly be extrapolated to the *in vivo *niche. For example, the addition of exogenous GDNF was not necessary, suggesting that GDNF is secreted from testis somatic cells, although exogenous GDNF and, to a lesser extent, EGF plus FGF2 enhanced cobblestone formation. Additionally, follicle-stimulating hormone (FSH), which has been reported to induce expression of GDNF in Sertoli cells, improved the formation of cobblestone colonies when used together with EGF plus FGF2. Interestingly, these findings suggest that other cytokines maintain the SSCs in the culture, since, in absence of exogenous FGF2, GDNF was able to increase the SSC population, likely due to FGF2 and/or additional FGF family molecules secreted by Sertoli cells. 

**Figure 1:**
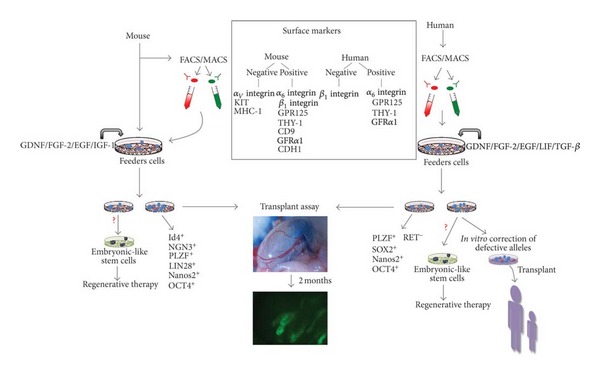
*In vitro* propagation of adult SSCs. SSCs are derived from the adult testis using somatic feeders and media containing diverse growth factors such as GDNF and FGF-2. In the case of human SSCs, a preenrichment sorting step (FACS or MACS) using previously identified surface marker, is critical for the successful expansion of SSCs. Mouse cultures established in such way can be maintained for over 1 year. Long-term expansion of SSCs *in vitro *is confirmed by analyzing the expression of molecular markers of spermatogonia. Furthermore, the number of stem cells expanded in the culture must be validated and quantified by an *in vivo *functional assay consisting of transplantation of SSCs into busulfan-treated recipient mouse testis. The fluorescent image corresponds to seminiferous tubules repopulated with donor GFP-positive cells. Potential clinical applications of SSCs include restoration of male fertility and/or *in vitro *correction of mutated alleles prior to transplant. Furthermore, *in vitro *SSCs can spontaneously reprogram to embryonic-like stem cells and could be used for regenerative therapy.

A certain amount of controversy remains in the field regarding the true identity and functionality of the proliferating cells that are enriched in long-term cultures of SSCs with different methods [7, 53, 67, 72]. Although it has been reported that feeder-free conditions could be employed [48] and that the proliferation of mouse SSCs *in vitro* is dependent on LIF instead of GDNF [73], most studies have shown that the use of both, mitotically inactivated somatic feeders cells and GDNF are critical for maintaining long-term self-renewal of SSCs *in vitro *(Figure 1).

The protocols to maintain mouse SSCs for long term in culture, together with what has been learned about culture conditions for human ES cells, have allowed the development of parallel strategies to propagate human SSCs. However, attempts to establish long-term cultures of human SSCs have been problematic. Among other genes, CD49f^+^, SSEA-4, GFR*α*1, GPR125, and PLZF are known to be expressed in human spermatogonia [5, 74–76]. Importantly, this gene expression information along with surface markers identified in spermatogonia from the primate testis and from other species has enabled the development of enrichment methods for putative human SSCs. For instance, human CD49f^+^ (*α*_6_ integrin) and SSEA^+^ germ cells preserved the ability to repopulate busulfan-treated testis in immunodeficient mice, suggesting not only that both populations are enriched in self-renewing SSCs but also that the niche is at least somewhat compatible between human and mouse [77]. However, as will be discussed further, nor SSEA-4 neither CD49f are considered specific marker for human spermatogonia, since they are also expressed either in somatic cells or in differentiating germ cells that coexpress markers of differentiation as well (e.g., KIT) [65]. Nevertheless, Chen et al. (2009) were able to maintain CD49f^+^ germ cells isolated from human fetal testes for two months using a combination of media containing a similar formulation to the one used to maintain mouse SSCs (GDNF, bFGF, and LIF) and human ES cell-derived fibroblast-like cells (hdFs) as feeder cells [78]. The human spermatogonial colonies derived in the culture expressed known markers associated with spermatogonia, like OCT4 [13, 49, 79]. 

A similar result was reported by Sadri-Ardekani et al. in 2009 for cells isolated from adult human testis from prostate cancer patients after orchiectomy [80]. The germline stem cell clusters that arose from testicular cell suspensions were cultured in media containing recombinant human epidermal growth factor (rhEGF), rhGDNF, and rhLIF. The SSCs were expanded up to four months, expressed spermatogonial markers, and were able to colonize recipient mouse testes [66]. In another study, the isolation of GPR125-positive spermatogonia from adult human testis resulted in an increase in undifferentiated cells after two weeks in culture in media containing human GDNF, LIF, EGF, and TGF and other factors that likely increase GDNF, FGF2, and TGF*β* signaling (GFR*α*1-Fc, NUDT6 and Nodal, resp.) (Figure 1) [76]. Similar to mouse SSCs, MAPK1/3 signaling was increased in GPR125-positive germ cells after two weeks in culture, revealing one molecular mechanism that may be involved in proliferation of human spermatogonia. Remarkably, the spermatogonia were obtained from deceased organ donors, a reasonable source from which to obtain human SSCs from healthy donors. A recent report using testicular tissue from patients with azoospermia identified genes differentially expressed between proliferating putative human SSCs *versus *the senescent human SSCs that resulted from human SSC-like cells after five passages in culture [81]. This yielded a list of potential genes related to proliferation of human germ cells.

## 4. Molecular Markers of Mouse and Human SSCs

Long-term expansion of SSCs *in vitro* cannot be fully realized until (1) stem cells are validated and quantified by the transplantation assay and (2) the identity of the expanded SSCs is confirmed through the use of molecular markers that distinguish them from spermatogonia in other stages of differentiation. Moreover, molecular markers expressed in SSCs are commonly employed using a variety of different techniques (i.e., immunohistochemistry or cell sorting strategies) to characterize and identify spermatogonia prior to culture. This becomes particularly relevant in the case of human SSCs, since, as opposed to mouse SSCs, fibroblasts are more easily established than SSCs from human testicular cultures without prior enrichment for spermatogonia [82]. 

The greatest limitation relies on the fact that SSCs are presumed to be a very rare population in the human testis, and their unequivocal identification has been extremely difficult to achieve. In fact, there are currently no specific markers expressed in mouse or human spermatogonia that are completely restricted to the pool of stem cells, although Oatley et al. (2011) have reported recently that Id4 expression is limited to the A_s_ pool in mouse spermatogonia [83]. As discussed previously, however, it remains unclear whether the *A*_s_ cells represent the only stem cell pool on the testis. Nevertheless, the results of many studies together have yielded a list of genes and surface markers proven to be expressed on SSCs, inspite of being not restricted to them, representing extremely valuable tools in SSC research as recently reviewed elsewhere [84, 85]. It was established very early that *β*_1_ integrin (CD29) and *α*_6_ integrin (CD49f) are expressed in mouse SSCs, while the KIT receptor tyrosine kinase and *α*_*v*_-integrin (CD51) are low or absent in undifferentiated spermatogonia and are considered to be markers of the transition to differentiating type A spermatogonia [86, 87]. Since *β*_1_-integrin and *α*_6_-integrin are located on the cell surface, they were promptly utilized in fluorescence-activated cell sorting (FACS) and magnetic-activated cell sorting (MACS) to enrich mouse SSCs from testicular tissue. Similarly, other surface markers were progressively identified as either expressed or absent, defining a surface phenotype for mouse spermatogonia [88–94]. Positive markers include THY-1 (CD90), CD9, GFR*α*1 and CDH1, while *α*_*v*_-integrin, KIT (CD117), MHC-1, and CD45 are negative or low. However, there are various technical reasons why surface expression does not guarantee that a given marker will be useful for stem cell enrichment (Figure 1). 

Recently, Kanatsu-Shinohara (2011) showed that the CD9^+^EPCAM^−/low^ population is more enriched for SSCs [95]. GPR125 is also a marker for undifferentiated spermatogonia in mouse [3]. In addition to surface markers, the expression of intracellular factors, including PLZF, LIN28, NANOS2, and OCT4, correlates with undifferentiated spermatogonia, although, once again, these are not restricted to the stem cell pool (Figure 1) [74, 96–100]. The zinc-finger RNA-binding protein NANOS2, for instance, regulates SSC maintenance and is expressed in *A*_s_,*A*_pr_, and some *A*_al_ [101]. Similarly, the promyelocytic leukemia zinc finger protein (PLZF) is a transcriptional repressor necessary for maintenance of germ cell lineage, generally associated with undifferentiated spermatogonia, including SSCs [102]. 

Several surface markers identified in mouse SSCs have been successfully tested in humans [75, 86]. For instance, THY-1 and GFR*α*1 were used to purify human spermatogonia by MACS. Similarly, He et al. (2010) employed GPR125 expression to isolate human spermatogonia and confirmed the expression of *α*_6_ integrin, THY-1, GFR*α*1, and PLZF in the sorted population [76]. Interestingly, only one or two spermatogonia per seminiferous tubule were estimated to express GPR125. On the other hand, neither *β*_1_ integrin, RET, nor NGN3 is considered to be markers for human spermatogonia [75, 85]. Additionally, molecular markers correlated with pluripotency (i.e., OCT4, NANOG, or SOX2) have been a detected in human spermatogonia, and it has been suggested that a subset of these cells exhibit the characteristics of pluripotent cells (Figure 1) [65, 103]. 

Other molecular markers in human spermatogonia have been recently identified and correlated with the different subpopulations established under the morphological criteria described earlier [104, 105]. For instance, only A_d_ spermatogonia express high levels of the exosome component 10 (EXOSC10), a feature linked to the immature state of the cell, while *A*_p_ and B spermatogonia share expression of Ki-67, which is associated with cell proliferation [106], and DMRT1, a protein that promotes differentiation-associated mitosis [107]. Furthermore, FGFR3 has been identified on the surface and in the cytoplasm of a subpopulation of rarely dividing type *A*_d_ spermatogonia that are also negative for Ki-67 and DMRT1. With such recently described molecular and functional markers have come newer theoretical models to explain the relationships between different subpopulations of human spermatogonia. For instance, it has been suggested that the nuclear differences observed in the *A*_p_ and *A*_d_ populations reflect stem cells in different stages of the cell cycle rather than spermatogonia in different stages of differentiation [26, 108].

## 5. Conclusions and Remarks

 While a variety of research applications and clinical uses of SSCs can be envisioned, the ability to manipulate SSCs *en masse* in the culture dish is a critical if not essential tool for most of such endeavors. Cell transplantation is a conceptually straightforward use of SSCs. For example, the *in vitro *correction of defective genes prior to transplant could be used either to restore male fertility or to prevent transmission of mutant alleles associated with genetic diseases. Pre pubertal human SSCs would be useful for chemotherapy-induced infertility arising from childhood cancers (Figure 1) [109]. However, it is also apparent that propagation of aberrant SSCs can lead to human diseases [110]. Therefore, it will be critical, in the future, to assess the potential risks and benefits associated with SSC-based therapy, including the possibility of propagating genetic or epigenetic abnormalities. With the proper knowledge base in place, including a good grasp of the intricacies of SSC self-renewal, only then can clinical strategies move forward successfully for the benefit of patients and their families.
